# Impact of Defined Risk Factors on Degree of Urinary Stress Incontinence and Sling Outcome: A Retrospective Cohort Analysis

**DOI:** 10.3390/jcm12165422

**Published:** 2023-08-21

**Authors:** Janine N. Frey, Mélanie Zellweger, Jörg Krebs, Corina Christmann

**Affiliations:** 1 Luzerner Kantonsspital Frauenklinik, 6000 Luzern, Switzerland; melanie.zellweger@luks.ch (M.Z.); corina.christmann@luks.ch (C.C.); 2 Clinical Trial Unit, Swiss Paraplegic Centre, 6207 Nottwil, Switzerland

**Keywords:** stress urinary incontinence, risk factor for severe SUI, success tension-free vaginal tape

## Abstract

Urinary stress incontinence is a distressing condition that has a severe impact on quality of life for most affected women. The insertion of the suburethral tension-free vaginal tape (TVT) is regarded as the gold-standard surgical treatment option. It is unclear whether all women with severe SUI benefit equally from TVT. Thus, the aim of our study was to identify risk factors for severe SUI and determine whether successful the resolution of incontinence after a TVT procedure was different in women with a higher degree of SUI. In total, 168 women were included in this retrospective cohort study. Women with severe SUI showed a significantly lower maximum urethral closure pressure (MUCP) (median 53 cmH_2_O in moderate vs. 39 cmH_2_O in severe, *p* = 0.001) and higher BMI (median 26.1 kg/m^2^ in moderate vs. 28.5 kg/m^2^ in severe, *p* = 0.045). Sonographic bladder neck funneling was detected significantly more often in women with severe SUI (27% in moderate vs. 57% in severe, *p* = 0.004). Lower MUCP and higher BMI were identified as significant predictors of severe SUI (*p* < 0.032). There was no difference in parity, age, functional urethral length and negative urethral stress pressure. Overall postoperative continence after the insertion of TVT was 91.9%. We found no significant difference in postoperative continence between women with severe vs. moderate SUI, suggesting that in our cohort the success of TVT was not significantly affected by the severity of SUI. In our cohort, low MUCP and high BMI were shown to be significant predictors of SUI severity. Nevertheless, treatment success of SUI with TVT did not differ substantially in women with more severe SUI.

## 1. Introduction

Urinary incontinence is a distressing and debilitating condition that has a severe impact on quality of life for most women who are affected by it. With a prevalence of up to 45%, stress urinary incontinence (SUI) is the most common type [1]. The condition can have huge social and economic impacts on the affected women and their families. 

SUI is defined by the International Continence Society (ICS) as “the complaint of any involuntary loss of urine on effort or physical exertion (e.g., sporting activities) or on sneezing or coughing”.

Over the years, various theories have been suggested to explain the pathophysiology of SUI. The earliest ones suggested that SUI occurs due to anatomical failure of urethral support. These were exemplified, for instance, by Enhorning G.’s hypothesis that SUI occurs due to reductions in urethral closure pressure during the transmission of abdominal pressure. Another theory emphasizes the intrinsic sphincter deficiency. More recently, researchers have attempted to combine the anatomical and functional hypotheses into a consolidated model with a multi-factorial etiology. For example, such a patient may be diagnosed with a disruption in the bladders and urethral supportive connective tissues, as well as weakened pelvic floor muscles (including the urethral sphincter), all leading to reduced urethral closure pressure and lower abdominal leak point pressure in a way that functionally results in SUI [1,2,3,4]. Mid-urethral support, provided by the pubo-urethral ligaments, also has an important role to play in maintaining continence and is the basis for the current use of minimally invasive mid-urethral slings (also called tension-free vaginal tape, TVT) in the treatment of SUI [1]. Considering the interplay between anatomical and functional factors, it is acknowledged that both structural and functional changes can contribute to the development of SUI, and that a combination of approaches may be needed for effective treatment.

First-line treatment options for SUI include conservative management, such as pelvic floor muscle training, weight loss, or continence pessaries. When first-line treatment modalities are unsuccessful, surgical interventions are offered. The surgical management options for SUI include mid-urethral slings (TVT), colposuspension, pubovaginal slings, single incision slings and bulking agents. Among these options, mid-urethral slings have been extensively studied and are considered to be the most researched surgical treatment for SUI [1]. According to a Cochrane analysis [1], long-term cure rates for TVT can reach up to 92%, with a low incidence of adverse events. Based on its efficacy, low degree of invasiveness and high level of safety, TVT are regarded as the first-line surgical choice for women affected by SUI and are considered the gold-standard treatment option [1,5]. TVT can be inserted via two main types of surgical approaches: the retropubic route and the transobturator route. In the retropubic method, the tape passes through the retropubic space blindly, either from the vagina to the abdomen or from the abdomen to the vagina. A bottom-to-top route is considered more effective than a top-to-bottom route [1]. In the transobturator route, the tape is inserted in a horizontal plane underneath the middle of the urethra through the obturator foramina. Comparing transobturator techniques involving medial-to-lateral and lateral-to-medial insertions; current evidence does not favor one approach over the other [1]. Regarding adverse events, the transobturator method is associated with a lower risk of bladder perforation and inducing bladder emptying disorders than the retropubic route. In contrast, the transobturator method is associated with a higher incidence of pain in the inner thighs and groin area, as well as more frequent vaginal injuries in the sulci region. Regarding erosion of the vaginal tape, there seem to be no significant differences between the two methods. Twelve months after the insertion of a transobturator TVT, patients have a higher risk of urethral perforation and chronic perineal pain [6].

When considering the cure rates, both retropubic and transobturator tapes show similar subjective and objective cure rates in the short term, medium term (one to five years), and long term (over five years). In the long term, subjective cure rates range from 43% to 92% in the transobturator group, and from 51% to 88% in the retropubic group [1]. 

However, it is unclear whether women with severe SUI equally benefit from retropubic or transobturator TVT as compared to women with mild or moderate SUI. 

The primary aim of this retrospective study was to identify established risk factors, such as urodynamic maximum urethral closure pressure (MUCP), functional urethral length, negative urethral stress pressure, sonographic bladder neck funneling (sonographic funneling), body mass index (BMI), parity, and age in our cohort, and to assess if they were different in women with more severe SUI. 

Secondary outcome measurement included an assessment of whether the severity of SUI had an impact on postoperative continence outcomes following the TVT procedure.

Adverse events, including postoperative voiding dysfunction after the insertion of a retropubic or transobturator TVT, were evaluated separately.

## 2. Materials and Methods

### 2.1. Study Design and Population

In this retrospective cohort study, we included all women undergoing a retropubic or transobturator TVT procedure at our tertiary referral hospital at the cantonal hospital of Lucerne from 2017 to 2020. 

All women with subjective and objective SUI who had undergone surgical intervention using TVT after unsuccessful conservative therapy were included in this study. We included premenopausal and postmenopausal women. Parity was included. Either the retropubic bottom-to-top or transobturator medial-to-lateral routes were applied. All women had at least one consultation preoperatively, where a comprehensive evaluation, including medical history, physical examination and often urodynamic assessment, was conducted. Six to eight weeks after the TVT insertion, another evaluation, including cough stress tests, residual urine assessment and sonographic examination, was performed and the success of the anti-continence measure was assessed.

Exclusion criteria were being aged under 18 years or if the follow-up was lost after the operation. 

The study was approved by the local ethics committee (BASEC ID 2022-00095). All women signed an informed consent form. 

### 2.2. Data Collection

Data were obtained and extracted from patients’ gynecologic hospital records. The data collected contained personal characteristics, such as age, parity, and BMI. 

### 2.3. Preoperative Assessments and Definitions

All women underwent a course of nine sessions of instructed pelvic floor physiotherapy before the continence surgery using sub-urethral sling insertion. Postmenopausal women were given a local estrogen supplement cream. 

The grading of the SUI was determined as follows: Grade I—loss of urine while coughing, sneezing or lifting something heavy; Grade II—loss of urine getting up or walking around; and Grade III—loss of urine in a lying position. We defined mild-to-moderate SUI as SUI Grade I/II and severe SUI as SUI Grade III. 

SUI was diagnosed subjectively, based on the patient’s reported loss of urine during activities, and objectively, using a clinical cough stress test in the lithotomy and standing position, with a minimal bladder filling of 300 mL. Alternatively, objective assessments were conducted via a pad weight test preoperatively and six to eight weeks postoperatively.

According to the International Continence Society (ICS) guidelines, further objective factors included urodynamic values, such as MUCP, functional urethral length and negative urethral stress pressure (MUCP is the maximum difference between the urethral pressure and the intravesical pressure, functional profile length is the length of the urethra, along which the urethral pressure exceeds intravesical pressure in women [7]). A perineal sonographic examination was performed to document bladder neck funneling preoperatively and postoperatively.

Most women underwent a full multichannel urodynamic assessment. Urodynamic studies included a conventional filling cystometry (with maximal bladder filling up to 500 mL) and a pressure flow study, in accordance with the recommendations of the ICS [7]. 

Residual urine was measured utilizing clean intermittent catheterization. An increased post-void residual (PVR) volume was defined as ≥100 mL [8].

MUCP was classified as low if it was less than the result of subtracting the patient’s age from 100. These assessments were carried out before and six to eight weeks after the TVT procedure. Postoperative success was determined as a negative cough test result and the patient’s affirmation that they were continent. 

The TVT procedure was performed by either a certified urogynecologist or urogynecologist trainees under supervision. All procedures were performed in an operating room. Retropubic TVT insertion was routinely chosen. However, in cases where a woman had undergone prior pelvic surgeries with cystoscopy that revealed a significant lateral deviation of the bladder, or had received prior pelvic radiation, a transobturator approach was chosen. For retropubic slings, the Gynecare TVT Exact System was applied using the bottom-to-top route. Cystoscopy was performed during the operation to detect possible bladder perforation. For transobturator slings, the Gynecare TVTTM Obturator System was applied using the medial-to-lateral route. No cystoscopy was performed when choosing the transobturator method. 

### 2.4. Statistical Analysis

The data were calculated as mean and standard deviation (SD), median with lower (LQ) and upper quartiles (UQ), or frequency. Differences between women with low-grade SUI and those with high-grade SUI were investigated using the unpaired Student’s *t* test, the Mann–Whitney U test, or Fisher’s exact test. The effect of risk factors (age, BMI, parity, functional urethral length, MUCP, negative urethral stress pressure, and sonographic bladder neck funneling) on the occurrence of severe stress incontinence (Grade III) was evaluated using binary logistic regression. The statistical analyses were performed using the SPSS software (Version 25, IBM, Somers, NY, USA) or in the R software environment (Version 3.4.0, Copyright 2017, The R Foundation for Statistical Computing, Vienna, Austria). A *p* value of ≤0.05 was considered significant.

## 3. Results

Overall, 168 women were eligible and included in this retrospective cohort study. Eight women were unavailable to return for the follow-up visit eight weeks after the TVT insertion due to the COVID-19 pandemic. In total, 153 women underwent retropubic TVT insertion and 15 underwent transobturator TVT insertion. Thirteen women had previously undergone incontinence operations (four transobturator TVT, three Burch procedures, three Bulkamid, two MiniArc slings, and one unknown sling). A total of 132 women were classified as SUI I/II, while 36 women were classified as SUI III.

The mean age was 55.4 ± 13.1 years (range 33–89 years). Mean BMI was 26.6 ± 5.4 kg/m^2^. Fifty-eight patients (38.2%) presented with a low MUCP, mean functional urethral length was 33.8 ± 10.1 mm, and sonographic funneling was observed in 53 patients (34.4%) (Table 1). Urodynamic studies were not performed in 16 patients, either because they had undergone prior urodynamics elsewhere or due to their refusal to undergo repeat urodynamics based on previous negative experiences. Functional urethral length and negative urethral stress pressure could not be measured in all cases due to technical difficulties and patient noncompliance, respectively.

In regard to the primary outcome, patients with SUI Grade III had a significantly (*p* = 0.001) lower median MUCP (39 cmH_2_O, 30/57 cmH_2_O) compared to those with Grade I/II (53 cmH_2_O, 38/74.5 cmH_2_O), a higher (*p* = 0.045) mean BMI (28.5 ± 5.9 kg/m^2^ vs. 26.1 ± 5.2 kg/m^2^), and showed significantly (*p* = 0.025) more frequent sonographic funneling (51.4% vs. 29.4%). There was no significant (*p* > 0.05) difference in parity, age, functional urethral length and negative urethral stress pressure between patients with SUI Grade I/II or SUI Grade III (Table 1). 

Both BMI und MUCP were significant (*p* < 0.032) predictors of severe SUI. An increase in BMI increased the odds of severe SUI, and an increase in MUCP reduced the odds of severe SUI (Table 2).

As a secondary outcome, we ascertained whether the severity of SUI influenced postoperative continence after the TVT procedure. Overall postoperative continence after the insertion of TVT was 91.9% (147 of 160), being 93.5% (116) in patients with SUI Grade I/II and 86.1% (31) in patients with SUI III (*p* = 0.17). These findings suggest that, in our cohort, the success of the TVT procedure was not significantly affected by the severity of SUI.

In regard to adverse events, we identified four women (2.3%) with transient voiding dysfunction postoperatively, which was resolved without intervention within six weeks. Two had SUI Grade II and two had SUI Grade III. All of them had undergone retropubic TVT insertion. One woman with SUI Grade III underwent tape revision four days after the retropubic TVT procedure. After the surgical intervention with the loosening of the tape, the voiding dysfunction was resolved. Two women, both having had retropubic TVT insertion and both with SUI Grade II, had persistent voiding dysfunction, leading to a revision operation where the tape was split. 

Two women showed tape erosion in the vagina and both presented with sympoms after the retropubic TVT procedure. No further adverse events were seen after performing a retropubic or transobturator TVT procedure. 

## 4. Discussion

This retrospective cohort study found that women with more severe SUI had a significantly lower MUCP, a higher BMI and presented sonographic funneling significantly more frequently than women with only mild-to-moderate SUI. Both MUCP and BMI were significant predictors of severe SUI, suggesting that these were the driving factors behind SUI severity in our cohort. 

In our study we found almost 40 percent of our patients to have low MUCP. These findings are similar to those in the current literature. DeLancey, J.O. et al. [9] described low MUCP as one of the strongest parameters associated with SUI, stating that a low MUCP plays an important role in the development of SUI. Fallah-Hassani et al. [2] also found that, among urethral closure pressure, measures of urethral support and other parameters, such as levator ani function and maximal cough pressure, MUCP was the strongest determinant of SUI. However, it is still unclear why some women have a lower MUCP than others. Research into the reason behind this may lead to new opportunities for prevention or to the development of different treatment strategies [9]. As age is a factor for reduced muscle mass, we could assume that a lower MUCP in our patients with SUI Grade III would also mean our patients with SUI Grade I/II were younger; however, there was no significant age difference in our cohort between SUI Grade I/II or Grade III. To our knowledge, there is no study to date that investigates how the MUCP has an impact on the severity of SUI. 

The second significant predictor of severe SUI values in our cohort was BMI scores with a 2.4 higher median in cases of severe SUI. Several studies have reported the association of SUI with higher BMI and weight [10,11,12,13]. Among these, the Norwegian EPINCONT study [14], which included almost 28,000 women, showed the clear dose–response effect of weight on the prevalence of urinary incontinence. Again, there are no studies examining the correlation between BMI and the severity of SUI. 

Interestingly, in our study, other known risk factors for SUI, such as sonographic bladder neck funneling, functional urethral length, negative urethral stress pressure, parity and age, did not seem to have an impact on the severity of SUI. 

Regarding our secondary outcome, the success of the treatment of SUI with TVT did not differ substantially in women with more severe SUI. The overall postoperative continence in our cohort after TVT insertion was 88.1%. These rates are similar to the rates reported in multiple studies [15,16,17,18]. Ford et al., in a Cochrane systematic review [1], showed a short-term subjective cure rate for retropubic and transobturator TVT of 62–98% and 71–97%, respectively (36 trials, 5514 women). The long-term subjective cure rates ranged from 43% to 92% in the transobturator group and from 51% to 88% in the retropubic group. 

In our cohort, even for women with risk factors for severe SUI, the gold-standard surgical treatment remained TVT insertion. 

Since our cohort predominantly underwent retropubic TVT (90.5%), it was not feasible to differentiate the success rates between the retropubic and transobturator methods within our cohort.

There are no studies investigating whether the severity of SUI has an impact on the success of the TVT procedure. 

The evaluation of adverse events in our cohort showed a low occurrence of such events. Three women (1.8%) had a surgical intervention due to persistent voiding dysfunction and two women (1.2%) suffered tape erosion, showing the TVT insertion to be a safe procedure overall. Ford et al., from the Cochrane systematic review [1], also reported a low overall rate of adverse events, reporting the rate of postoperative voiding dysfunction to be 5.53% in 37 trials with 6200 participants, and the rate of vaginal tape erosion to be 2.09% in 31 trials with 4743 participants. Other adverse events, such as bladder or urethral perforation, de novo urgency, groin pain, and major vascular/visceral injury, did not occur in our cohort.

The first limitation of our study is its retrospective observational design. More prospective studies that include risk-factor-guided analysis are needed. The second limitation is the short follow-up period for assessing postoperative continence rates, with follow-up required only six to eight weeks post-TVT insertion.

## 5. Conclusions

In conclusion, this study highlights that lower MUCP and higher BMI are significant predictors of SUI severity that can potentially be used to guide future prevention and treatment strategies. TVT is a safe procedure with a high success rate, even in women with severe SUI. Further studies are needed to validate these findings. These should preferably include randomized control trials with control groups and a longer follow-up period.

## Figures and Tables

**Table 1:** Patients’ characteristics, subdivided by SUI grade.

	SUI Grades I–II		SUI Grade III		*p*	All	
		*n*		*n*			*n*
Age (years)	54.8 ± 13.0	132	57.7 ± 13.4	36	0.83	55.4 ± 13.1	168
Parity * (*n*)	2 (2/3)	128	3 (2/3.5)	33	0.24	2 (2/3)	161
BMI (kg/m^2^)	26.1 ± 5.2	127	28.5 ± 5.9	36	0.045	26.6 ± 5.4	163
MUCP (cmH_2_O) *	53 (38/74.5)	117	39 (30/57)	35	0.001	50 (36.25/67.75)	152
Functional urethral length (mm)	33.8 ± 9.8	112	34.0 ± 11.0	35	0.94	33.8 ± 10.1	147
Negative urethral stress pressure	93 (84.5%)	110	32 (91.4%)	35	0.6	125 (86.2%)	145
Sonographic funneling (*n*/%)	35 (29.4%)	119	18 (51.4%)	35	0.025	53 (34.4%)	154

* Median value, with lower and upper quartiles.

**Table 2:** Binary logistic regression regarding risk factors predicting severe SUI.

Predictors	End of Primary Rehabilitation		
	*p*	Exp(B)	95% CI Exp(B)
Age	0.82	0.99	0.96–1.03
Parity	0.89	1.03	0.70–1.50
BMI	0.03	1.09	1.01–1.18
MUCP	0.011	0.97	0.95–0.99
Functional urethral length	0.61	1.01	0.97–1.06
Sonographic funneling	0.08	2.22	0.91–5.42
Negative urethral stress pressure	0.74	1.28	0.30–5.49

Exp(B): odds ratio predicted by model. CI: confidence interval.

## Data Availability

For data supporting reported results please consult with the corresponding author.
